# Geschlossene/minimal-invasive Repositionstechniken an der oberen und unteren Extremität in der Kindertraumatologie

**DOI:** 10.1007/s00064-025-00892-y

**Published:** 2025-02-28

**Authors:** Kai Ziebarth, Theddy Slongo

**Affiliations:** Abteilung für Kinderorthopädie/Kindertraumatologie, Kinderchirurgische Universitätsklinik, Inselspital, Freiburgstr., 3010 Bern, Schweiz

**Keywords:** Fraktur, Reposition, Kind, Gips, Konservative Therapie, Fractures, Fracture reduction, Child, Surgical casts, Conservative treatment

## Abstract

**Behandlungsziel:**

Stabile Reposition einer Fraktur in eine tolerable Stellung. Verhinderung einer erneuten Abkippung der Fraktur. Vermeidung von invasiven Operationen respektive Minimierung der Verwendung von Fremdmaterial.

**Indikationen:**

Frakturen des Kindes an der oberen und unteren Extremität.

**Kontraindikationen:**

Gelenkfrakturen. Trümmerfrakturen. Offene Frakturen.

**Technik:**

Korrekte Analgesie/Narkose, um die Intervention für das Kind so angenehm wie möglich durchzuführen. Je nach Lokalisation und Frakturmuster ist eine indirekte Reposition durch die Ruhigstellung, z. B. Blount-Schlinge bei einer suprakondylären Humerusfraktur, oder eine direkte manuelle Reposition der Fraktur mit anschließender Gipsruhigstellung notwendig. Unterstützend können zur Retention perkutane Kirschner-Drähte/Schrauben oder ein externer Fixateur verwendet werden.

**Weiterbehandlung:**

Im Anschluss an jede Reposition mit Gipsruhigstellung ohne weitere Fixation sollte eine Stellungskontrolle 5 bis 7 Tage nach Manipulation durchgeführt werden. Je nach Stellungsverhältnissen ergibt sich dann das weitere Procedere. Wurde eine Osteosynthese durchgeführt, ist keine Stellungskontrolle notwendig, sondern lediglich die Konsolidationskontrolle 3 bis 6 Wochen (altersabhängig) postoperativ.

**Ergebnisse:**

Durch geschickte Repositionen und meist minimal-invasive Verfahren, können in der Kindertraumatologie viele Frakturen geschlossen oder sogar ohne eine Osteosynthese behandelt werden.

## Abstract (en)

**Objective:**

Stable reduction of pediatric fractures to the accepted position. Prevention of recurrent dislocation or loss of reduction to avoid invasive surgery.

**Indications:**

Pediatric fractures of the upper and lower extremities.

**Contraindications:**

Joint fractures, comminuted fractures, open fractures.

**Technique:**

A comfortable environment for the child as well as sufficient pain management is of highest importance for successful treatment. Depending on the location of fracture or fracture pattern, indirect reduction (e.g. by cuff and collar), or direct manual reduction is applied with or without fixation of the fragments (screws, Kirschner wire, external fixator).

**Postoperative management:**

Follow-up radiograph 5–7 days after closed reduction. In case of retention with hardware, a consolidation control with radiograph 3–6 weeks postintervention (depending on the age of the patient) is appropriate.

**Results:**

Introduction of closed reduction techniques makes pediatric fracture treatment feasible without open interventions or need of osteosynthesis.

## Vorbemerkungen

In der Kindertraumatologie ist die Art der Frakturbehandlung sowohl vom Alter des/der Patienten/in als auch vom betroffenen Skelettsegment abhängig. Gerade bei Frakturen nahe potenter Wachstumsfugen muss nicht auf eine anatomische Reposition gedrängt werden. Ziel der Behandlung ist es, alters- und lokalisationsabhängig eine altersentsprechende tolerable Achsabweichung zu erzielen [4, 6, 7, 9]. Aufgrund der biomechanischen Beschaffenheit des Knochens bei Kindern (dickes Periost, höhere Elastizität) ist gleichzeitig die Inzidenz von instabilen oder Trümmerfrakturen deutlich geringer. Somit entstehen in der Kindertraumatologie viele Frakturmuster, welche durch geschlossene Repositionstechniken behandelt werden können.

Ebenso ist die Stabilität nach Reposition einer Fraktur bei Kindern aufgrund des kräftigen Periostes deutlich besser, verglichen mit einem erwachsenen Patienten/in. In Fällen, wo durch die alleinige Reposition keine stabile Situation erzielt wird, können zur Unterstützung perkutan gesetzte Kirschner-Drähte, kanülierte Schrauben oder ein externer Fixateur eingesetzt werden. Die Indikation zu einer offenen Reposition mit großen Operationszugängen kann daher bei Kindern im Vergleich zur Erwachsenentraumatologie zurückhaltend gestellt werden.

Die Voraussetzung für eine erfolgreiche Reposition ist das Verständnis der Frakturmorphologie und des Entstehungsmechanismus der Fraktur, da manchmal lediglich die dislozierenden Kräfte umgekehrt werden müssen, um eine Fraktur wieder adäquat zu reponieren.

In diesem Artikel werden häufige Repositionstechniken vorgestellt, welche typischerweise in der Kindertraumatologie angewendet werden. Aus Kapazitätsgründen können nicht alle minimal-invasiven Repositionstechniken, wie z. B. die in der Kindertraumatologie häufig angewandte „elastisch stabile intramedulläre Marknagelung“ (ESIN), beschrieben werden. Hier verweisen wir auf die gängige Literatur [1].

## Behandlungsprinzip

Geschlossene oder minimal-invasive Behandlung einer Fraktur beim Kind, unter Berücksichtigung der alters- und lokalisationsbedingten Toleranzgrenzen. Vermeidung von unnötigen stationären Aufenthalten und/oder invasiven Therapiemaßnahmen.

## Vorteile

- Geschlossene Behandlung ohne große Operationsnarbe
- Kein Blutverlust
- Keine Fadenentfernung
- Metallentfernung selten (perkutane K‑Drähte in Analgosedation)
- Ambulante Behandlung meist möglich
- Geringe Kosten

## Nachteile

- Gefahr der erneuten Frakturabkippung bei unzureichender Reposition oder Retention
- Schmerzhafte Manipulation bei insuffizienter Schmerzbehandlung/Analgosedation
- Meistens keine funktionelle Nachbehandlung möglich (Ausnahme Fixateur externe oder nach perkutanen Schrauben)

## Indikationen

Die Anwendung der hier genannten kindertraumatologischen Therapieansätze, insbesondere der Einbezug des Remodeling, ist verständlicherweise nur möglich, solange an dem betroffenen Knochen die Epiphysenfugen noch offen sind, respektive ein Restwachstum zu erwarten ist. Die oben erwähnten biomechanischen Vorteile des jugendlichen Skeletts (dickeres Periost etc.), sind sicherlich auch noch darüber hinaus beim jungen Erwachsenen vorhanden und können auch hier genutzt werden. Der Übergang zur Erwachsenentraumatologie ist daher fließend und je nach Geschlecht und Entwicklung des/der Patienten/in individuell zu beurteilen. In der eigenen Klinik werden in der Kinderklinik Patienten/innen bis zum Abschluss des 16. Lebensjahres behandelt, jedoch werden uns nicht selten Patienten/innen > 16 Jahre aufgrund der radiologisch fehlenden Skelettreife (offene Epiphysenfugen) von den Kollegen der Erwachsenentraumatologie zur weiteren Behandlung zugewiesen.

### Obere Extremität

- Dislozierte Frakturen des distalen Vorderarmes beim Kind (Salter Harris 1 und 2, Grünholzfrakturen, Bowing)
- Fingerfrakturen/Metakarpalfrakturen
- Suprakondyläre Humerusfrakturen
- Humerusschaftfrakturen
- Subkapitale Humerusfrakturen

### Untere Extremität

- Eminentia-intercondylaris-Frakturen
- Unterschenkelschaftfrakturen
- Dislozierte distale Unterschenkelfrakturen beim Kind (Salter Harris 1 und 2)
- Zehenfrakturen

### Relative Indikationen

- Femurfrakturen beim Kleinkind < 3 bis 4 Jahre (proximal und Schaft)
- Instabile (lange Schräg- und Spiralfrakturen) Unterschenkelschaftfrakturen
- Dislozierte/angulierte Radiushalsfrakturen (schwierig)
- Distale Femurfrakturen
- Unterarmschaftfrakturen (wegen der geringen Abweichungstoleranz meist TEN-Osteosynthese)

## Kontraindikationen

- Intraartikuläre Frakturen
- Trümmerfrakturen
- Offene Frakturen
- Schaftfrakturen mit wenig Abweichungstoleranz, z. B. proximaler Unterarmschaft/Femurschaft
- Suprakondyläre Humerusfrakturen (AO Typ III & IV)

## Patientenaufklärung

- Art und Weise der Schmerzbehandlung sowie allenfalls Narkose (Generalanästhesie, Regionalanästhesie, Lachgas)
- Möglichkeit der erneuten Dislokation mit Möglichkeit der Reintervention
- Notwendigkeit zur Osteosynthese, wenn keine stabile Retention in akzeptabler Stellung möglich ist
- Offene Reposition, wenn geschlossene Reposition nicht erfolgreich

## Vorbereitungen

- Analyse des Frakturtyps sowie der Dislokationsrichtung
- Korrekte Positionierung des Bildwandlers zur freien Durchleuchtung der Extremität
- Einrichten des Bildschirmes des Bildwandlers, sodass während der Manipulation freie Sicht auf den Monitor besteht
- Sicherstellung einer korrekten a.-p. und seitlichen Projektion der Extremität
- Allenfalls Instruktion des Personals zur korrekten Positionierung des Bildwandlers, ggf. Drehen der Strahlenquelle um die Extremität herum, um eine korrekte Darstellung in 2 Ebenen zu erhalten, ohne die Extremität zu viel bewegen zu müssen
- Vorbereitung des Gipsmateriales und Anlegen des Gipsstrumpfes vor der Reposition zur problemlosen Gipsanlage nach Reposition
- Korrekte Instruktion des Hilfspersonals bezüglich Haltens der Extremität respektive Anreichung von Gipsmaterial
- Genügend anwesendes Hilfspersonal (an der unteren Extremität 2 bis 3 Personen), um insbesondere nach der Reposition den Gips applizieren zu können, ohne dass es zu einer erneuten Fehlstellung kommt

## Instrumentarium

- Bei der Behandlung der oberen Extremität allenfalls Finger-Strips/Extensionshülsen für die Finger installieren
- Bildwandler
- Gipsmaterial inklusive Gipssäge etc.
- Kirschner-Drähte
- Kanülierte Schrauben
- Fixateur externe

## Anästhesie und Lagerung

- Wahlweise je nach Alter und Compliance der/des Patienten/in Vollnarkose vs. Regionalanästhesie, Lachgas, Analgosedation (Ketamin)
- Sowohl bei der Reposition der oberen als auch der unteren Extremität ist der/die Patient/in meist in Rückenlage gelagert
- An der oberen Extremität erleichtern die Finger-Strips/Extensionshülsen die Positionierung des Armes zur Gipsanlage, auch bei Frakturen am distalen Radius mit geringer Dislokation, wo keine Reposition notwendig ist

## Technik

**Abb. 1:**
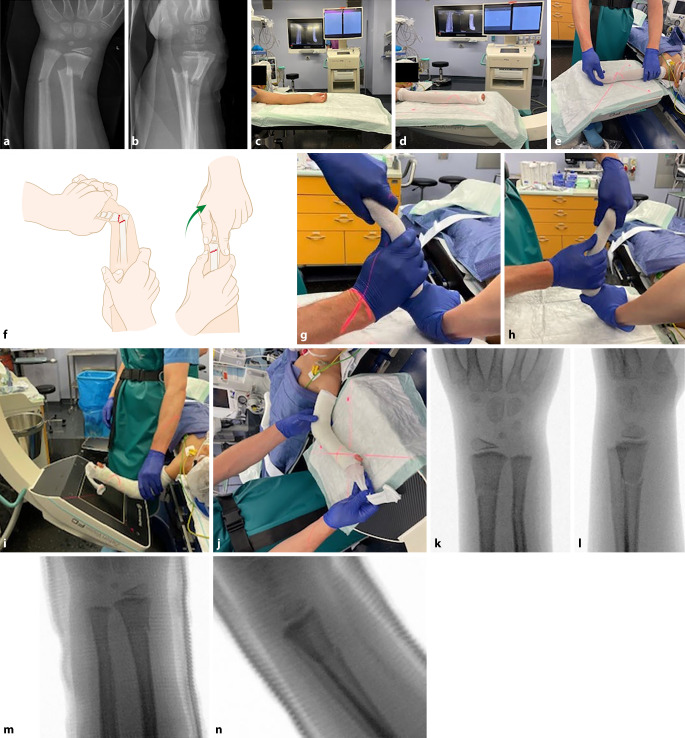
**a,** **b** Stark nach radiodorsal dislozierte metaphysäre Radiusfraktur bei einem 5‑jährigen Knaben. **c–e** Lagerung des Patienten entweder auf Armtisch (**c**) oder direkt auf dem Topf des Bildwandlers (**d,** **e**). Wichtig ist, dass der Operateur während der Reposition freie Sicht auf den Bildschirm hat. Es empfiehlt sich, bereits vor der Reposition den Gipsstrumpf anzulegen, um nach der Reposition den Arm nicht mehr stark zu manipulieren und den Gips schnellstmöglich anzupassen. **f–h** Durch Traktion respektive Dislozieren des distalen Frakturfragmentes nach dorsal können die Hinterkanten und somit die Fraktur stabil aufeinander gestellt werden. Cave: Eine pure Flexion des distalen Frakturfragments führt zu einer verbleibenden Verkürzung, da die Hinterkanten nicht aufeinander stehen, sodass eine Redislokation im weiteren Verlauf die Folge ist. **i–n** Nach vollzogener Reposition Gipsanlage und radiologische Kontrolle unter dem Bildwandler. Am besten gelingt dies durch eine Positionierung des Bildwandlers um den Patienten, sodass der Arm nicht exzessiv manipuliert werden muss. Die Reposition wird vor Gipsanlage (**k,** **l**) sowie nach Gipsanlage dokumentiert (**m,** **n**)

**Abb. 2:**
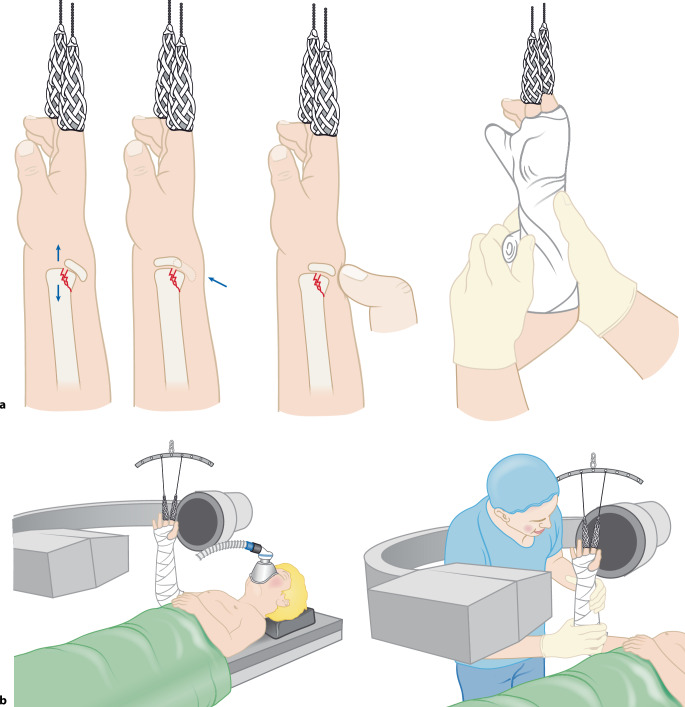
Alternativ Reposition mittels Finger-Strips/Extensionshülsen für die Finger. **a** Zunächst Traktion durch Aushängen mittels Finger-Strips/Extensionshülsen für die Finger. Je nach Alter des Patienten kann eine perorale Analgesie in Kombination mit Lachgas oder auch eine Kurznarkose auf der Notfallstation, z. B. mit Ketamin, erfolgen. Es gelten dieselben Regeln für die Reposition wie in Abb. 1. Ist die Fraktur nur disloziert und nicht verkürzt, kann durch Druck von dorsal die Fraktur in Position gebracht werden. Kontrolle der Reposition in 2 Ebenen. Nun kann die seitliche Verschiebung ebenfalls noch korrigiert werden. Bei unzureichender Reposition muss das Manöver wiederholt werden. **b** Bei nur gering über der Toleranzgrenze dislozierten Frakturen im metaphysären Bereich kann auch der Arm lediglich in Finger-Strips/Extensionshülsen für die Finger (je nach Größe des Patienten mit 0,5–1 kg Gewicht) zum Vereinfachen der Gipsanlage aufgehängt werden. Hier ist meist nur eine perorale Analgesie notwendig. *Cave: Bei SH1-Frakturen sollte der Patient in Vollnarkose und relaxiert sein, um bei der Manipulation Scherkräfte auf die Epiphysenfuge und damit eine Schädigung zu vermeiden*

**Abb. 3:**
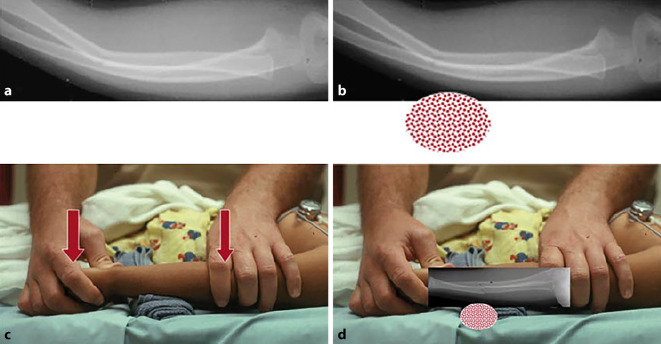
**a,** **b** Grünholzfraktur am Vorderarm. **c,** **d** Apex der Fehlstellung auf Unterlage positionieren, um die Fraktur über das „Hypomochlion“ gegenbrechen zu können

**Abb. 4:**
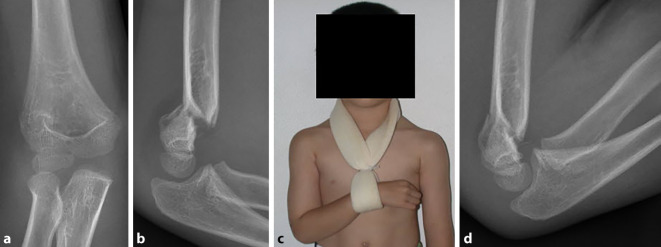
**a,** **b** Suprakondyläre Humerusfraktur (Extensionstyp) bei 4‑jährigem Knaben. **c** Positionierung des Armes in Blount-Schlinge in Flexion. Meistens kann der Ellbogen initial nur bis etwa 80–90° flektiert werden. Deutliche Extensionsfehlstellung ohne Rotationskomponente (hintere Kortikalis aligniert). **d** Nach 3 bis 5 Tagen, wenn der Schmerz nachgelassen und die Schwellung abgenommen hat, sollte dann die Schlinge nachgezogen werden, um den Ellbogen etwa 120–140° zu flektieren. Es empfiehlt sich, den Eltern anzuraten, vor der Konsultation zum Nachziehen der Schlinge dem Kind daheim Schmerzmittel zu geben. In diesem Beispiel zeigt sich eine Flexion des Ellbogens nach dem Nachziehen der Schlinge von 140 Grad. Dies lässt trotz des Verbleibes einer diskreten Extensionsstellung des distalen Fragmentes eine gute Funktion nach Ausheilung erwarten, auch wenn sich die Restfehlstellung nicht vollständig remodellieren wird (in sagittaler Ebene bis ca. 6. Lebensjahr; [2]). Ein signifikanter Rotationsfehler liegt nicht vor, was an der im Verlauf quasi undislozierten dorsalen Kortikalis zu erahnen ist. Wichtig ist, dass bei den radiologischen Verlaufskontrollen mit meist nicht optimaler seitlicher Röntgenaufnahme eine schräg abgebildete Frakturzone nicht mit einem Rotationssporn verwechselt wird

**Abb. 5:**
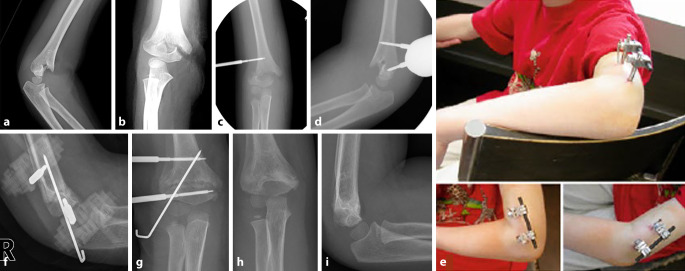
**a,** **b** Falls die geschlossene Reposition nicht funktioniert, kann mithilfe eines radialen eingebrachten Fixateurs extern die Fraktur unproblematisch reponiert werden, ohne dass eine offene Reposition notwendig ist. **c,** **d** Hierfür wird unter Bildwandlerkontrolle ein Schanzpin (meist 3,0/4,0 Seldrill) über eine Stichinzision in den distalen Humerus ca. 1 cm proximal der Frakturlinie eingebracht. Danach wird das distale Frakturfragment exakt seitlich eingestellt und hier ebenfalls perkutan ein Schanzpin eingebracht. **e** Die beiden Pins werden über eine (oder über 2 Stangen mit Tube-to-Tube zur einfacheren Reposition in allen Ebenen) Querstange verbunden. Der distale Schanzpin wird als Joystick verwendet und die Fraktur zunächst auf Länge reponiert. Anziehen der Fixateurbacken. Kontrolle der Reposition unter dem Bildwandler in beiden Ebenen. Durch Lösen der Backen und vorsichtige Manipulationen kann dann die definitive Reposition erfolgen. Zur Verbesserung der Rotationstabilität wird im Anschluss nach korrekter Reposition ein 2,0-mm-Kirschner-Draht perkutan von radial eingebracht (aus [8]). **f,** **g** Konsolidationskontrolle nach 4 Wochen. **h,** **i** Kontrolle nach Entfernung des Fix-Ex in Lachgasanalgesie

**Abb. 6:**
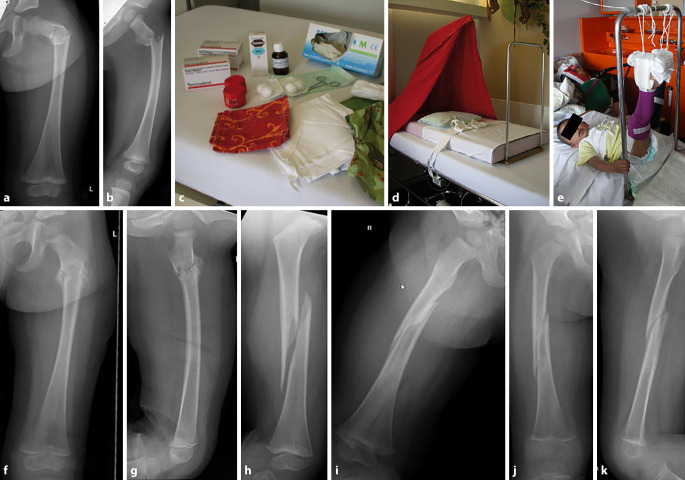
**a,** **b** Knabe, 4 Jahre. Dislozierte sub-/intertrochantäre Femurfraktur. Overhead-Extension-Prinzip: Ausschaltung der die Fraktur dislozierenden Muskeln (Hüftflexoren, insbesondere M. iliopsoas), sodass sich die Fraktur passiv anatomisch einstellt. Ausführliche Information der Eltern über den Sinn (Vermeidung einer Operation) und die Technik der Behandlung. **c,** **d** Vorbereiten der Klebeextension. Applikation der Klebeextension unter suffizienter Analgesie (keine Narkose notwendig). Vorbereiten des Bettbogens unter der Matratze (mobil). **e** Vorsichtiges „Aufhängen“ der Beine am Bettbogen, bis das Gesäß freischwebt. Nach initialer Hospitalisation von 1 bis 2 Tagen kann das Kind dann nach Hause entlassen werden, da der Bettbogen unter der Matratze mobil ist, sodass das Kind mit einem Bettentransport transportiert werden kann (entsprechend individueller Infrastruktur und Organisation des Krankenhauses, ansonsten ggf. stationäres Setting). **f,** **g** Nach 3 bis 4 Wochen nehmen die Eltern das Kind von der Overhead-Extension und bringen es mit liegenden Verbänden an den Oberschenkeln in die Sprechstunde. Zeigt sich eine anatomische Konsolidation der Fraktur, kann die Klebeextension entfernt werden. Dies ist am einfachsten, wenn die Eltern das Kind daheim in eine Badewanne (sofern vorhanden) setzen, sodass die Verbände aufweichen und dann problemlos entfernt werden können. Dies kann aber je nach Wunsch der Eltern auch in der Poliklinik durch das Pflegepersonal erfolgen. **h–k** Beispiel Kind 2,5 Jahre Femurschaftfraktur. Gleiche Therapie. Konsolidation nach 3 Wochen (**j,** **k**)

**Abb. 7:**
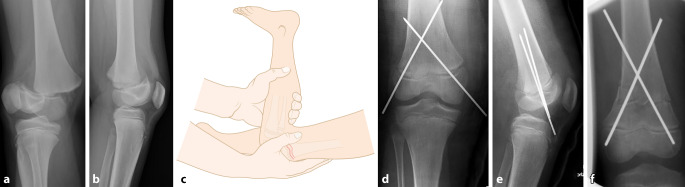
**a,** **b** Knabe, 12 Jahre, dislozierte Salter-Harris-1-Fraktur distales Femur. **c** Nach Intubationsnarkose Positionierung des Patienten in Bauchlage, um durch Traktion und Flexion im Kniegelenk eine einfache Reposition zu erreichen. Cave: Da diese Fraktur meist bei größeren/schwereren Kindern vorkommt, sollte eine gute Relaxation des Patienten erfolgen. Die Reposition und Retention in Hyperflexion des Kniegelenkes (analog zur Hyperflexion bei der Reposition suprakondylärer Humerusfrakturen) sind in Bauchlage einfacher. Es kann dann durch perkutan eingebrachte Kirschner-Drähte die Fraktur stabilisiert werden. **d,** **e** Es empfiehlt sich, die Kirschner-Drähte von distal dorsal nach ventral proximal zu setzen, um eine Verletzung der Gefäß-Nerven-Strukturen (v. a. medial) zu verhindern. Entweder werden die Drähte distal mediolateral überstehen gelassen (**d,** **e**) oder nach proximal retrograd ausgeleitet (anderer Patient, **f**), bis die Drahtenden distal unter dem Knochen verschwinden (vermeidet die Kommunikation der Drähte mit dem Kniegelenk!)

(Abb. 1, 2, 3, 4, 5, 6, 7, 8 und 9)

**Abb. 8:**
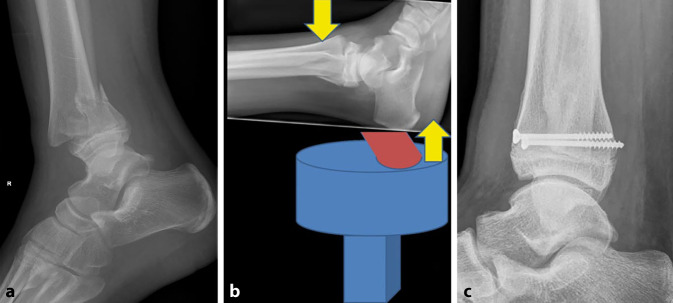
**a** Knabe, 8 Jahre. Dislozierte SH-2-Fraktur distale Tibia. **b** Lagerung des Fußes direkt auf dem BV. Reposition durch Traktion und ventralen Druck auf die Tibia. Falls Reposition nicht anatomisch, empfiehlt es sich, leichte bis mäßige Schläge mit dem Handballen gegen die distale Tibiakante auszuüben, dadurch „rutscht“ das distale Fragment nach ventral und „rastet ein“. Meist stabile Situation. Osteosynthese nicht notwendig. Im Zweifel perkutane Schraube von anterior nach posterior (**c**)

**Abb. 9:**
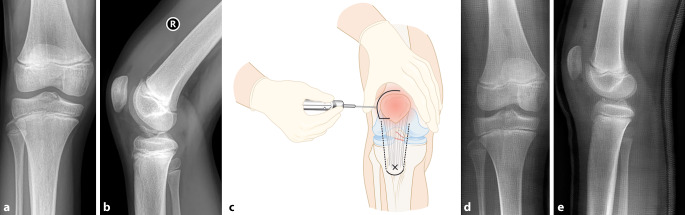
**a,** **b** Mädchen, 9 Jahre. Trauma mit dem Fahrrad. Dislozierte Eminentia-intercondylaris-Fraktur (Typ 2 nach Meyer und Mckeever). **c** Nach Gabe von Analgetika und/oder Auflage von lokalbetäubendem Pflaster kann das Knie von anterolateral punktiert werden, um den Hämarthros zu entfernen und gleichzeitig lokales Anästhetikum zu applizieren. Nun kann das Knie schmerzfrei extendiert und eingegipst werden. **d,** **e** Zeigt die radiologische Kontrolle eine akzeptable Stellung des Eminentiafragmentes, kann konservativ behandelt werden. Ist dies nicht der Fall, z. B. wenn ein Teil des Meniskus im Frakturspalt liegt und die Reposition des Fragmentes verhindert, ist eine meist arthroskopisch assistierte Reposition und Fixation des Fragmentes indiziert

## Besonderheiten

In der Kindertraumatologie muss das Vorgehen je nach Alter und Compliance des Kindes geplant und v. a. auch mit den Eltern abgesprochen werden. Es obliegt dem behandelnden Arzt/Aerztin, den Eltern (dem Kind) die Vorteile der entsprechend gewählten Therapie zu erklären, auch wenn dies auf den ersten Blick für die Eltern als unmöglich erscheint, diese Behandlung mit ihrem Kind zu akzeptieren (z. B. Overhead-Extension bei Femurfrakturen).

Trotzdem kommt es aber nicht selten vor, dass aufgrund des ausdrücklichen Wunsches der Eltern eine Operation mit Narkose und Osteosynthese durchgeführt wird, anstatt konservativ zu behandeln.

## Postinterventionelle Behandlung

- Stellungskontrolle 5 bis 7 Tage postoperativ/nach Manipulation ohne Osteosynthese (Ausnahme: Bei Femurfrakturen an Overhead-Extension wird keine Stellungskontrolle durchgeführt)
- Gips für 3 bis 6 Wochen (gemäß Alter des/der Patienten/in)
- Konsolidationsröntgen gipsfrei nach 3 bis 6 Wochen (gemäß Alter)
- Eine Funktionskontrolle 3 bis 4 Wochen nach Gipsentfernung kann je nach lokaler Infrastruktur beim Kinderarzt erfolgen.
- Physiotherapeutische Maßnahmen, sind in der Regel nur in Ausnahmefällen, wenn auch nach längerer Zeit keine gute Funktion spontan zurückgewonnen wurde (ca. 4 bis 6 Wochen nach Gipsentfernung), notwendig.
- Ist die Fraktur mit einer tolerablen Fehlstellung ausgeheilt, welche aber noch von außen sichtbar ist, werden je nach Alter der/des Patienten/in 6 bis 12 monatliche klinische Verlaufskontrollen empfohlen. Dies v. a. zur Beruhigung der Eltern. Hierdurch lassen sich unnötige Arztkonsultationen mit möglicherweise unnötigen Korrekturversuchen durch nicht kindertraumatologisch erfahrene Ärzte vermeiden.

## Fehler, Gefahren, Komplikationen und ihre Behandlung

- Schmerzen im Gips durch zu eng angelegten Gips:
  Gipsspaltung oder sogar Neuapplikation des Gipses (Aufhängen in Finger-Strips/Extensionshülsen für die Finger)
- Erneutes Abkippen der Fraktur, außerhalb der tolerablen Grenze bei Stellungskontrolle durch zu locker angelegten Gips: je nach Ausmaß der Abkippung Reintervention mit ggf. bei instabilen Verhältnissen Notwendigkeit zur Osteosynthese. Gipskeilung
- Erneutes Abkippen der Fraktur in tolerabler Grenze bei Stellungskontrolle durch ungenügende Reposition oder zu locker angelegten Gips: Gipswechsel, da evtl. zu locker, ggf. nach 5 bis 7 Tagen erneute Stellungskontrolle. Alternativ: Gipskeilung zur Vermeidung des weiteren Abrutsches oder zur Verbesserung der Stellung, wenn Fraktur außerhalb der tolerablen Grenzen. In seltenen Fällen erneute Reposition notwendig
- Schmerzen/Unruhe bei Behandlung. Z. B. Nichttolerieren der Overheadextension bei Femurfraktur: Umstellen auf neues Behandlungsverfahren (Beckenbeingips vs. TEN-Osteosynthese)

## Ergebnisse

Die meisten der oben erwähnten Repositionstechniken und Behandlungen wurden in den letzten 40 Jahren erfolgreich in der Institution der Verfasser angewendet [2, 3]. Es kam zu nur sehr wenigen Komplikationen oder Verfahrenswechseln. Schaut man sich in der Literatur die prozentualen Angaben für Remanipulationen nach geschlossener Reposition von distalen Vorderarmfrakturen, welche mit etwa 40 % der Frakturen beim Kind den größten Anteil ausmachen, an, so werden Zahlen zwischen 7 und 35 % genannt [5]. Diese breite Spanne der prozentualen Angaben deutet darauf hin, dass diese anspruchsvolle Technik ein gewisses Maß an Erfahrung und auch manuellen Skills erfordert, welche jedoch problemlos erlernt werden können. Sind sich Arzt/Ärztin dessen bewusst, sind positive Behandlungsergebnisse mit niedrigen Reinterventionsraten zu erwarten.

Allerdings führen der gesellschaftlichen Wandel und die damit verbundenen immer höheren Ansprüche der Eltern an die Mediziner/innen (möglichst sichere und definitive Behandlung ohne für das Umfeld sichtbare Veränderungen wie Restfehlstellung trotz sicheren Remodelings) in den letzten Jahrzehnten dazu, dass trotz größeren Aufwands, wie z. B. zusätzliche Narkose, stationärer Aufenthalt etc., der operative Weg inklusive Osteosynthese der konservativeren Behandlung (geschlossene Reposition mit ggf. tolerabler Restfehlstellung) vorgezogen wird.

## Data Availability

Die in dieser Studie erhobenen Datensätze können auf begründete Anfrage beim Korrespondenzautor angefordert werden.
